# Mechanisms Underlying Cognitive Impairment Induced by Prenatal Alcohol Exposure

**DOI:** 10.3390/brainsci12121667

**Published:** 2022-12-04

**Authors:** Ahmad Alhowail

**Affiliations:** Department of Pharmacology and Toxicology, College of Pharmacy, Qassim University, Al Qassim 51452, Saudi Arabia; aalhowail@qu.edu.sa

**Keywords:** alcohol, prenatal alcohol exposure, neurogenesis, cognitive impairment

## Abstract

Alcohol is one of the most commonly used illicit substances among pregnant women. Clinical and experimental studies have revealed that prenatal alcohol exposure affects fetal brain development and ultimately results in the persistent impairment of the offspring’s cognitive functions. Despite this, the rate of alcohol use among pregnant women has been progressively increasing. Various aspects of human and animal behavior, including learning and memory, are dependent on complex interactions between multiple mechanisms, such as receptor function, mitochondrial function, and protein kinase activation, which are especially vulnerable to alterations during the developmental period. Thus, the exploration of the mechanisms that are altered in response to prenatal alcohol exposure is necessary to develop an understanding of how homeostatic imbalance and various long-term neurobehavioral impairments manifest following alcohol abuse during pregnancy. There is evidence that prenatal alcohol exposure results in vast alterations in mechanisms such as long-term potentiation, mitochondrial function, and protein kinase activation in the brain of offspring. However, to the best of our knowledge, there are very few recent reviews that focus on the cognitive effects of prenatal alcohol exposure and the associated mechanisms. Therefore, in this review, we aim to provide a comprehensive summary of the recently reported alterations to various mechanisms following alcohol exposure during pregnancy, and to draw potential associations with behavioral changes in affected offspring.

## 1. Introduction

Alcohol (ethanol) is perhaps the most commonly used and socially accepted psychoactive substance. Alcohol consumption is highly addictive, and evidence indicates that it can cause serious systemic side effects, such as heart and lung diseases, and increase the risk of cancer and susceptibility to some infectious diseases [1,2]. More recently, alcohol use was also identified as a risk factor for dementia and cognitive decline [3,4]. Recent studies showed that 10% of pregnant women are exposed to alcohol worldwide [5,6]. Alcohol consumption is not recommended during pregnancy due to the risk of cognitive dysfunction in offspring [7], and studies provided undeniable evidence that alcohol exposure during pregnancy can lead to mental retardation in children [8,9]. Fetal alcohol spectrum disorders (FASDs) are an umbrella term used to refer to the neurobehavioral and physical impacts of prenatal alcohol exposure, and fetal alcohol syndrome (FAS) is one of the most severe consequences in this spectrum of disorders. FASD is a serious global public health concern, and its effects can persist through to adulthood. Its incidence ranges from 1% to 20% among children [10], and in younger school-aged children, its incidence can be as high as 20–50 cases per 1000 children [11]. The approximate incidence of FASDs in the general population was roughly estimated at about 1–2 cases per 1000 individuals [12], while the approximate incidence of FAS is 9–10 cases per 1000 individuals [13]. According to the United States Centers for Disease Control and Prevention, the incidence of FASD in the U.S. is relatively high (1.5–2.0 cases/1000 births) [14], and two studies in Europe showed that the incidence of FASDs is 23–47 cases per 1000 individuals [15] and 40 cases per 1000 individuals [16]. Importantly, the occurrence of FASDs was linked to the socioeconomic status of the mother, with maternal low education level and low income associated with a higher incidence of FASDs [11].

Clinical studies showed alterations in cognitive functions, such as the intelligence quotient score, of children with prenatal alcohol exposure [17,18,19]. In animal studies, the offspring of pregnant rats exposed to alcohol also showed cognitive impairment in fear conditioning and spatial memory tasks [14,20,21]. FASDs are commonly associated with a range of cognitive deficits that include memory impairments, specifically intrusion errors and confabulation, difficulties with strategic manipulation of information to improve recall, difficulties with the initial encoding of information, mixed expressive–receptive language disabilities, visual–motor integration and visual–perceptual deficits, learning disabilities, poor response inhibition, impulsiveness, perseverative behavior, impaired executive functioning, and deficiency in adaptive skills [22]. Prenatal alcohol exposure during the first trimester is associated with deficits in learning, and short- and long-term memory, specifically in the verbal domain [23]. Further, a prospective study in the UK found that alcohol consumption during the second and third trimesters of pregnancy was strongly related to an increased risk of mental health problems, especially inattention and hyperactivity, in girls at 47 months, and in both boys and girls at 81 months [24]. In New Zealand, higher rates of depression were reported among children exposed to alcohol in the prenatal period than those among unexposed children [25]. However, some studies have found only weak associations between alcohol consumption and neurobiological deficits. For example, in a Danish national study, exposure to binge drinking was only weakly associated with impaired behavioral and emotional development measured at age 7 [26]. Further, a study on children in rural Burkina Faso demonstrated that maternal alcohol consumption was associated with poorer neuropsychological performance in children aged 6–8 years, but it could not establish a causal relationship between the two [27]. Such inconsistencies may be a result of differences in study populations, definitions of alcohol consumption, and assessment methods. Despite these differences, studies so far indicate that alcohol consumption during pregnancy can, overall, alter neurodevelopmental processes in the offspring during embryonic development, ultimately leading to cognitive impairment and affecting neurobehavioral performance [28,29].

Alcohol that crosses the placental barrier to reach the fetus [30] is not effectively metabolized because the liver does not mature until the late stage of development; this results in an increase in the alcohol concentration in fetal circulation [31,32]. With regard to its mechanism of action, alcohol binds the gamma-aminobutyric acid (GABA) receptor, which is a major inhibitory neurotransmitter in the brain [33,34]. Through this interaction, alcohol acts as a GABA receptor agonist in central nervous system (CNS) tissues such as the amygdala, hippocampus, cerebellum, and cortex, thereby affecting brain development in the fetus [35,36]. The other reported mechanisms by which prenatal alcohol exposure causes cognitive impairment include the disruption of synaptic plasticity and long-term potentiation (LTP) [14], and the disruption of neurogenesis [37], mediated by the effects of alcohol on protein kinases [14,38,39], and mitochondrial dysfunction [5,6]. However, there are still some gaps in the knowledge regarding the mechanisms underlying the cognitive effects of alcohol on the fetus. Moreover, to the best of our knowledge, there are very few recent reviews that focused on the molecular mechanisms underlying the cognitive effects of prenatal alcohol exposure in offspring. Therefore, in this review, we discuss the recent studies on this topic in order to provide a comprehensive understanding of the mechanisms by which prenatal alcohol exposure induces cognitive impairment in offspring, and lay a basis for potential strategies that can be used to ameliorate these effects.

## 2. Behavioral Studies on the Effects of Prenatal Alcohol Exposure

Behavioral tests are used to evaluate cognitive function and memory formation in both animals and humans [40]. Some common tests used to assess memory function in animal models include the Y maze, novel object recognition, Morris water maze (MWM), radial arm maze, and contextual fear conditioning tasks [14,41,42]. Contextual fear conditioning tests involve a training session in which animals receive an electric shock that is preceded by a tone. In the test session, the period of time for which the animal remains frozen after the delivery of the shock is compared with that of animals who are exposed to the tone without the shock [14]. Studies using this technique showed that animals exposed to alcohol during pregnancy exhibited a shorter freezing time than that of control animals that had not been exposed to alcohol; this indicates that prenatal alcohol exposure causes cognitive impairment in offspring [14,43].

The Y-maze test is commonly used to evaluate spatial memory [44]. The maze is composed of three arms connected to each other via a chamber at the center. In this test, animals are allowed to explore two arms in an exposure period that ends with a certain cue. The third arm (novel) remains closed during the exposure session and is opened only during the test session. Behavior is evaluated by counting the number of entries and the total time spent in the novel arm in the test animals and comparing the findings with those of nontreated or nondiseased animals. Animals exposed to alcohol in the prenatal period made more spatial reference memory errors in the Y- and T-maze tests than the control animals did; further, spatial working memory errors were more common in males than in females [45,46].

The MWM consists of a large round tub of water with a platform that is hidden from view with the addition of powdered milk to the water [47]. In MWM tests, animals are allowed to swim, find the hidden platform, and stand on it during the training period. During the experiment, the animal is placed in the maze and allowed to find the hidden platform. The time spent on the platform and the distance covered by the animals while locating the hidden platform are tracked and recorded. The time spent reaching the platform is considered to be an indicator of learning and memory ability [47,48]. Studies conducted MWM tests and showed that the offspring of animals exposed to ethanol during pregnancy had impaired cognitive function compared to that of animals who had not been exposed to ethanol [49,50]. The virtual water maze, which is analogous to the MWM in animals, is used to test spatial memory in humans. In this test, children try to learn the location of a hidden platform in a virtual water pool through a sequence of learning trials. In the navigation task, their ability to locate the hidden platform is tested. Similar to the results obtained with rodents exposed to alcohol during pregnancy, the performance of children who had been exposed to alcohol in the prenatal period was poor and indicative of cognitive impairment [51].

Studies using the radial arm maze showed memory impairment in prenatal alcohol-exposed animals compared with control nonexposed animals [52]. In this test, animals are trained in a maze consisting of eight equally spaced arms connected at the center, and a cup with or without food is placed at the end of each arm. After the training session, animals are tested for their ability to identify the arm containing food, and the number of errors is counted and compared with the results of control nontreated or nondiseased animals. Although several studies showed a higher error rate among animals exposed to alcohol in the prenatal period than that of nonexposed controls [53,54], differences in cognitive function between these groups were not observed in other studies that employed this test. These discrepancies may be accounted for by differences in the time point at which the animals were evaluated, as some studies conducted these tests on postnatal day 90 (P90) and other conducted the tests between P26 and P60 [54].

The findings from the experimental animal models discussed above have also been reported in human subjects. For example, one systematic review reported impaired verbal and visual-spatial episodic memory performance in school-aged children and adolescents with prenatal alcohol exposure [55]. Further, another systematic review found that prenatal exposure to alcohol has long-term cognitive, behavioral, social, and emotional developmental consequences in adolescents that vary according to the amount and timing of exposure in utero [56].

To conclude, the findings of behavioral studies in both animal models and humans prove that prenatal exposure affects learning and memory functions in the offspring.

## 3. Effect of Prenatal Alcohol Exposure on Body and Brain Weight

Body weight is regulated by genetic, metabolic, and environmental factors. However, under certain circumstances, changes in homeostasis and behavior can cause weight gain or loss. Body weight is usually a reflection of health status, and alterations in body weight are a marker of brain function [57]. For example, an increase in body weight due to a high-fat diet can result in the development of Type 2 diabetes, which eventually causes cognitive impairment [58,59] and a reduction in blood cerebral flow [60,61,62]. In addition, an increase in BMI and obesity can cause a reduction in cerebral blood flow as a result of the increase in body weight, which can cause alterations in brain function [63]. Studies also revealed that neurological diseases, such as Alzheimer’s and Parkinson’s diseases, can cause body weight reduction [63,64]. In addition, neurological studies indicated that brain size is a marker of brain and cognitive function [65,66]. For instance, brain size, particularly in terms of the size of grey and white matter, is reduced in cancer patients following chemotherapy [67] and is thought to represent one of the mechanisms underlying cognitive dysfunction following chemotherapy [68]. Accordingly, studies on the effects of prenatal alcohol exposure on offspring revealed a reduction in body weight, and brain size and weight, particularly in the amygdalae and hippocampus, which may affect brain function and lead to cognitive impairment [43,69,70]. Thus, the cognitive effects of prenatal alcohol exposure may involve a reduction in body as well as brain weight.

## 4. Effect of Prenatal Alcohol Exposure on Neurogenesis

Neurogenesis, the process by which new neurons are generated, occurs in the brain in the embryonic period and continues into adulthood [71]. However, in adulthood, neurogenesis is restricted to specific regions of the brain such as the hippocampus and dentate gyrus [72,73,74]. During neurogenesis, neural stem cells proliferate and differentiate into mature neurons that integrate with other neurons to form the neuronal circuitry [75,76]. Neurons communicate with other neurons via electrochemical interactions at the synapse [77]. Neurogenesis and synaptogenesis in the hippocampus are vital for acquired learning and memory formation [78]. Previous studies revealed that a reduction in hippocampal neurogenesis results in memory impairment [37,79,80]. In addition, neurogenesis is reduced in the hippocampus of the offspring of rats exposed to alcohol in the prenatal period [81]. Thus, it is speculated that impaired neurogenesis is a potential mechanism of prenatal alcohol exposure-induced cognitive dysfunction in offspring. In fact, one study that used human brain organoids as an experimental model found that alcohol exposure impaired neurogenesis in these organoids [82]. This is supported by the findings of another study that used human cortical organoids and reported that alcohol exposure had temporally dependent effects on proliferation, cell cycle, and apoptosis [83].

## 5. Effect of Prenatal Alcohol Exposure on Synaptic Plasticity

Learning and memory processes occur in the hippocampus, which is a part of the limbic system of the brain that is responsible for memory formation. In these processes, the synaptic structure is altered, and its shape is modified, so that more receptors are present in the synaptic cleft; this is known as synaptic plasticity. Synaptic plasticity is also applied at the cellular level of synaptic neurons to describe the communication that occurs during memory formation. The synaptic response is based on the release of neurotransmitters from presynaptic neurons and the response of receptors on the postsynaptic neurons, and any alterations in these processes lead to a reduction or even abolition of LTP. Measurements of LTP and long-term depression (LTD) can be used to determine the strength and weaknesses of synapses in a certain neuronal pathway. Electrophysiological studies using in vitro and in vivo models have revealed changes in synaptic activity in the hippocampus following behavioral tasks, thereby confirming the link between synaptic change and memory formation. Further, studies showed a reduction in the LTP of the offspring of pregnant rats exposed to alcohol [14], indicating that prenatal exposure to alcohol can lead to a persistent reduction in LTP in offspring that subsequently results in cognitive impairment. This is supported by observations in human cortical organoid experiments that demonstrated that alcohol exposure affected glutamatergic synaptic development, and neural network formation and activity [83]. Human studies have not measured LTP in offspring with prenatal alcohol exposure, but one study reported that prenatal alcohol exposure is associated with specific EEG correlates in infants and children [84]. Further, another study on children with FASD found a reduction in theta frequency band power on EEG recordings taken during a memory-guided task [85]. These results could reflect changes in synaptic plasticity that occur as a result of prenatal alcohol exposure, but they need to be confirmed.

## 6. Effect of Prenatal Alcohol Exposure on Mitochondrial Function

The mitochondrion is the power house of the cell and is involved in many cellular processes, such as oxidative phosphorylation, ATP production, β-fatty acid oxidation, the Krebs cycle, and the regulation of apoptosis [86]. In particular, mitochondrial DNA (mtDNA) is important for enzymes and signaling pathways required for energy production within the mitochondria [42,87,88]. Alcohol exposure during pregnancy can alter mitochondrial morphology and function in the offspring [5,6]. Further, studies also showed that ethanol exposure can alter mtDNA via an increase in 8-hydroxydeoxyguanosine incorporation and mtDNA single-strand breaks, and a reduction in mtDNA content, which lead to mitochondria dysfunction [89,90,91]. Additionally, fetal ethanol exposure can increase lipid peroxidation, DNA damage, and reactive oxygen species generation, and reduce ATP synthesis and storage [92]. As mitochondrial cellular respiration plays a critical role in regulating cellular and cognitive functions, alterations in the process of cellular respiration can impact cognitive function. Interestingly, prenatal alcohol exposure impairs cognitive function by reducing the activity of mitochondrial complex I and complex IV [93,94,95]. Therefore, these mitochondrial complexes warrant further study from the viewpoint of understanding the mechanisms of cognitive impairment induced by prenatal alcohol exposure and identifying targets for treatment.

## 7. Activation of the GABA Receptor in the CNS of the Fetus in Response to Prenatal Alcohol Exposure

GABA receptors are expressed from Day 12 of the embryonic period and play an essential role in brain development and the general regulation of brain functions, including learning and memory formation [36,36,96]. GABA receptors are highly expressed in the CNS, predominantly in the amygdala, hippocampus, cortex, cerebellum, and hypothalamus [97]. They exist in two distinct forms, namely, GABA_A_ and GABA_B_ [98,99]. GABA_A_ receptors are ligand-gated chloride ion channels, whereas GABA_B_ receptors are G-protein-coupled receptors [100]. They are composed of five subunits, and different types of GABA receptors [101] are distinguished by the composition of these five subunits. The most common type of GABA receptors are composed of two α subunits, two β subunits, and one γ subunit. GABA receptors are involved in synaptic transmission and facilitate learning and memory processes, and GABA receptor activation appears to inhibit neuronal firing and the release of other neurotransmitters, such as glutamate and dopamine, in the CNS.

In experimental animal models, GABA receptor hyperactivation inhibits the release of glutamate and dopamine, thereby reducing the levels of other neurotransmitters linked to behavior modulation through its ability to regulate cognitive function. Further, studies showed that alterations in the expression of GABA receptors in animals impairs memory function [102,103], and prenatal alcohol exposure can chronically alter brain development and function in the offspring [104]. One study on cortical plate samples from fetal and infant brains demonstrated insufficient and delayed production of GABAergic interneurons in the ganglion during the two first trimesters of pregnancy and their delayed incorporation into the laminar structures of the frontal cortex, as well as mispositioning of GABAergic and calretininergic interneurons throughout fetal life [105]. Thus, alterations in GABA expression may be a potential mechanism underlying the cognitive dysfunction caused by prenatal alcohol exposure. This means that GABA receptors may be a viable target for the treatment of cognitive problems caused by prenatal alcohol exposure.

## 8. Effect of Prenatal Alcohol Exposure on Protein Expression and Activity

**Figure 1:**
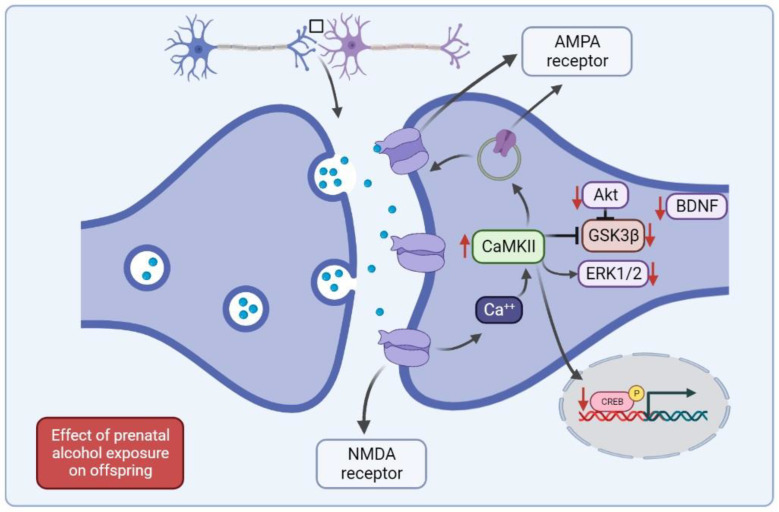
Diagrammatic representation of the cellular mechanisms of cognitive impairment induced by prenatal alcohol exposure. AMPA = α-amino-3-hydroxy-5-methyl-4-isoxazole propionic acid, NMDA = *N*-methyl-d-aspartate, CaMKII = calcium calmodulin dependent kinase II, Akt = protein kinase B, GSK3β = glycogen synthase kinase 3 beta, ERK1/2 = extracellular regulated kinase ½, BDNF = brain-derived neurotrophic factor, CREB = cAMP-response element binding protein.

Proteins play a vital role in regulating many physiological functions at the cellular level, including cell structure, metabolism, and differentiation, and synaptic plasticity and learning and memory [106,107,108]. With regard to neuronal stimulation and memory formation, proteins facilitate signal transduction from receptors to activate different protein kinases [109,110]. These signaling pathways regulate the gene expression and protein synthesis required to modify ion channel properties and ion channel density in the synapses [111,112,113]. The expression of several proteins in the hippocampus is altered during hippocampal-dependent tasks, and these proteins include brain-derived neurotrophic factor (BDNF), calcium calmodulin dependent kinase II (CaMKII), extracellular regulated kinase ½ (ERK1/2), cAMP-response element binding protein (CREB), α-amino-3-hydroxy-5-methyl-4-isoxazole propionic acid (AMPA) receptor, and N-methyl-d-aspartate (NMDA) receptor [114,115,116,117]. In addition, the phosphorylation of protein kinase B (PKB or Akt) and glycogen synthase kinase 3 beta (GSK3β) is altered. These proteins play an essential role in neuronal signaling, signal transduction, and neurotransmitter synthesis and release, thereby facilitating learning and memory processes [112,118] (Figure 1).

Akt is a downstream protein of phosphoinositide-3-kinase (PI3K), and the Akt/PI3K pathway functions as a prosurvival pathway [119]. Akt phosphorylation plays a key role in regulating cellular processes, such as survival, proliferation, and apoptosis, and the neurological pathways involved in learning and memory formation [120]. Akt phosphorylation at Ser^473^ leads to GSK3β inhibition through phosphorylation at Ser^9^, thereby reducing glycogen synthesis and increasing the energy available for memory formation. The phosphorylation of Akt and GSK3β was reduced in the brains of offspring exposed to alcohol during the prenatal period, and as a result, energy consumption, signaling, and trafficking of other receptors, such as glutamate AMPA and NMDA receptors, were impaired. AMPA and NMDA receptors play a key role in regulating memory function. These receptors are composed of tetramers of four subunits assembled in different combinations [121]. AMPA receptors consist of GluR1 to GluR4 subunits, while NMDA receptors contain GluN1, GluN2, and GluN3 [122,123]. Under normal physiological conditions, AMPA receptors are impermeable to calcium, while NMDA receptors are permeable [124]. The calcium impermeability of AMPA receptors is due to the presence of the GluR2 subunit that contains Mg^2+^ [124,125]. The presence of GluR2 in the synaptic AMPA receptors prevents Ca^++^ influx, thus reducing excitotoxicity [126,127]. In GluR2-knockout mice, increased levels of GluR1 in AMPA receptors resulted in greater Ca^++^ influx into neurons, and this led to epileptic seizures and thus apoptosis [127]. Therefore, alterations in the surface expression of these subunits could alter memory formation. The surface expression of GluR2 was increased in the hippocampus of rats exposed to alcohol and nicotine during pregnancy; this indicates that cognitive dysfunction in offspring is caused by GluR2 overexpression at the synaptic surface [14]. In addition, alterations in the subunit composition in NMDA receptors can alter cognitive function, and the GluN1 and GluN3 composition of synaptic NMDA receptors is increased following prenatal alcohol exposure. Thus, changes in the subunit composition of AMPA and NMDA receptors may play a role in the mechanism by which cognitive function is impaired in offspring with prenatal alcohol exposure, and these receptors may be viable targets for treatment.

BDNF belongs to a family of neurotrophins that control many physiological functions, including cell survival, differentiation, neurogenesis, and synaptogenesis [128]. With regard to memory function, BDNF plays a critical role in regulating learning and memory processes and synaptic plasticity in the brain. The underlying mechanism involves BDNF-induced activation of tropomyosin-related kinase B (TrkB) receptors, which belong to the tropomyosin-related kinase family. Previous studies revealed that BDNF mRNA expression was increased in the hippocampus of animals after training in the MWM, radial arm maze, and contextual fear conditioning tests [114,128,129]. However, a reduction in BDNF mRNA expression is associated with memory impairment in dopamine transporter-knockout mice [130]. Accordingly, the intrahippocampal injection of BDNF improved the memory function of animals in MWM tests, while the administration of anti-BDNF antibodies led to memory impairment [131,132]. BDNF also regulates the expression and function of various proteins necessary for synaptic plasticity and cognitive function through the modulation of the TrkB receptor [133]. When BDNF binds to the extracellular region of this receptor, it enhances tyrosine kinase activity, leading to autophosphorylation by ATP [134]. The activation of the TrkB receptor activates other pathways, such as the mitogen-activated protein kinase (MAPK) and PI3K/AKT pathways, and regulates CaMKII [135], all of which play essential roles in the regulation of cognitive function [136]. BDNF expression is reduced following prenatal alcohol exposure, and this reduction in BDNF levels could result in alterations in other signaling pathways that ultimately cause cognitive impairment [14]. These findings indicate that alcohol exposure during pregnancy reduces the cognitive function of offspring via inhibition of Akt, GSK3β, and BDNF activities.

CaMKII is a protein kinase that is involved in calcium signaling in eukaryotic cells [137]. This protein is activated by increase in intracellular calcium, and it phosphorylates various proteins that are involved in mobilization of synaptic vesicles, modulation of ion channels, regulation of gene expression, regulation of learning and memory processes, and LTP [138,139]. CaMKII is present downstream of NMDA receptors, and activation by Ca^2+^ influx through NMDA receptors causes autophosphorylation of CaMKII [140]. There is considerable evidence that alterations in CaMKII activity in the brain can lead to cognitive impairment [141,142,143]. Further, the overactivation of CaMKII can induce neurotoxicity and, thus, apoptosis [144]. Similarly, a reduction in CaMKII activity induces cognitive dysfunction. In addition, CaMKII activation can phosphorylate the AMPA-GluR1 subunit at the Ser^831^ residue and lead to the mobilization of the GluR1 subunit to the synaptic membrane [113]. CaMKII also plays a critical role in LTP induction and maintenance [145]. In rats with prenatal alcohol exposure, CaMKII activities were significantly increased compared to the control levels [146]. These findings indicate that CaMKII induces apoptosis in the brain, which could be a potential mechanism of cognitive impairment in offspring exposed to alcohol in the prenatal period.

ERK1/2 signaling is required for normal physiological functions [147]. In the brain, ERK1/2 plays a vital role in regulating neuronal development, function, and synaptic plasticity [148]. ERK1/2 is activated through its phosphorylation, and this can enhance signaling pathways that are involved in mediating neuroprotection during oxidative stress responses [38,149] and facilitate memory consolidation [39]. In addition, several lines of evidence showed that ERK1/2 phosphorylation is required for formation of short-term and long-term memory as well as mediating LTP and LTD [150,151]. Likewise, ERK1/2 can regulate the activities of transcriptional factors, such as CREB and BDNF, which are well-established as vital factors in learning and memory processes [152]. Therefore, alterations in ERK1/2 activity can alter synaptic plasticity and memory formation [153,154]. With regard to the association of ERK1/2 with prenatal alcohol exposure, ERK1/2 phosphorylation is reduced in offspring with alcohol exposure during pregnancy [43,155,156]. Thus, a reduction in ERK1/2 phosphorylation may play an important role in memory impairment in offspring.

CREB is a transcription factor that plays a critical role in regulating several physiological functions [157]. CREB is activated by several signaling pathways, such as the insulin, PI3K/AKT, and MAPK pathways [158,159]. This activation of CREB occurs through phosphorylation at the Ser^133^ residue [152,160]. Once CREB is activated, it is transported from the cytoplasm to the nucleus, where it binds the CRE promoter region, which is involved in gene transcription, translation, and protein synthesis [152,161]. Recent studies revealed that activation of CREB plays a role in hippocampal-dependent tasks by enhancing synaptic plasticity, LTP, and memory formation. In contrast, the reduced activity of CREB induces LTD by causing synaptic AMPA receptor endocytosis [162,163,164]. Interestingly, studies reported that CREB phosphorylation is reduced in the offspring of mothers exposed to alcohol during pregnancy [165,166,167]; this might represent another mechanism of cognitive impairment caused by prenatal alcohol exposure. Thus, prenatal alcohol exposure may induce cognitive impairment through inhibition of CREB activities, and this may cause a reduction in GluR1-containing AMPA receptors in the synaptic surface and induce LTD.

Thus, various proteins have been implicated in the mechanisms underlying the cognitive effects of prenatal alcohol exposure, namely, Akt, GSK3β, NMDA and AMPA receptors, BDNF, CaMKII, ERK1/2, and CREB. In the future, it would be interesting to explore their potential as treatment targets in offspring affected by cognitive dysfunction caused by alcohol exposure.

## 9. Conclusions

**Figure 2:**
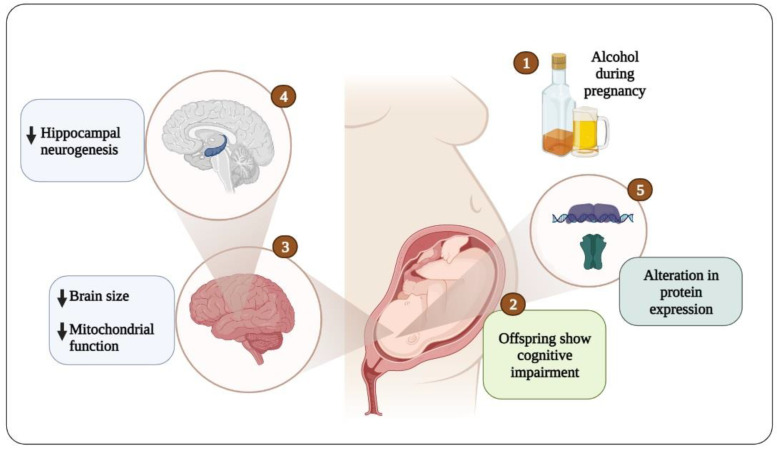
Conceptual diagram of the onset of cognitive impairment due to embryonic alcohol exposure.

Prenatal alcohol exposure caused cognitive impairment via different mechanisms in humans and experimental animal models, and these include the disruption of synaptic plasticity, LTP, neurogenesis, and mitochondrial dysfunction (Figure 2). Alcohol has the ability to pass through the placental and blood–brain barriers to access the fetal brain and activate GABA receptors, thereby affecting development and leading to irreversible changes in brain function. Prenatal exposure to alcohol reduces the expression and function of proteins, such as phosphorylated Akt, phosphorylated GSK3β, BDNF, phosphorylated ERK1/2, phosphorylated CREB, and phosphorylated CaMKII, and alters the composition of the AMPA and NMDA receptor subunits, and this leads to an increase in glutamate GluR2 surface expression. These changes are associated with mitochondrial dysfunction, and decreased neurogenesis in the hippocampus and cortex, and ultimately lead to cognitive dysfunction in the offspring. Thus, these proteins and their pathways would be interesting topics of investigation in future studies on the treatment of cognitive dysfunction caused by alcohol exposure.
